# PACS-1 variant protein is aberrantly localized in *Caenorhabditis elegans* model of PACS1/PACS2 syndromes

**DOI:** 10.1093/genetics/iyae118

**Published:** 2024-07-20

**Authors:** Dana T Byrd, Ziyuan Christina Han, Christopher A Piggott, Yishi Jin

**Affiliations:** Department of Neurobiology, School of Biological Sciences, University of California, San Diego, La Jolla, CA 92093, USA; Department of Neurobiology, School of Biological Sciences, University of California, San Diego, La Jolla, CA 92093, USA; Department of Neurobiology, School of Biological Sciences, University of California, San Diego, La Jolla, CA 92093, USA; Department of Neurobiology, School of Biological Sciences, University of California, San Diego, La Jolla, CA 92093, USA

**Keywords:** embryonic development, neuronal development, synaptic function, Furin-binding domain, WDR-37, protein sorting, rare disease mutations

## Abstract

PACS (phosphofurin acidic cluster sorting) proteins are known for their roles in sorting cargo proteins to organelles and can physically interact with WD40 repeat-containing protein WDR37. PACS1, PACS2, and WDR37 variants are associated with multisystemic syndromes and neurodevelopmental disorders characterized by intellectual disability, seizures, developmental delays, craniofacial abnormalities, and autism spectrum disorder. However, the functional effects of syndromic variants at the cellular level remain unknown. Here, we report the expression pattern of *Caenorhabditis elegans* orthologs of PACS and WDR37 and their interaction. We show that cePACS-1 and ceWDR-37 colocalize to somatic cytoplasm of many types of cells and are mutually required for expression, supporting a conclusion that the intermolecular dependence of PACS1/PACS2/PACS-1 and WDR37/WDR-37 is evolutionarily conserved. We further show that editing in PACS1 and PACS2 variants in cePACS-1 changes protein localization in multiple cell types, including neurons. Moreover, expression of human PACS1 can functionally complement *C. elegans* PACS-1 in neurons, demonstrating conserved functions of the PACS–WDR37 axis in an invertebrate model system. Our findings reveal effects of human variants and suggest potential strategies to identify regulatory network components that may contribute to understanding molecular underpinnings of PACS/WDR37 syndromes.

## Introduction

PACS (phosphofurin acidic cluster sorting) protein family was first identified based on protein binding to a C-terminal acidic cluster motif within Furin protease (Wan *et al*. 1998) and is generally known to function in mediating cargo sorting or return to the trans-Golgi network (Youker *et al*. 2009; Thomas *et al*. 2017). PACS proteins are conserved from invertebrates to human (Thomas *et al*. 2017). They are multidomain proteins with N-terminus binding to Furin and C-terminal domains involved in autoregulation. Vertebrate genomes express 2 paralogs of PACS, and invertebrates generally have only 1 PACS homolog. Studies in multiple cell lines have revealed that interactions of acidic cluster motifs with the Furin-binding region (FBR) of PACS1 and its paralog, PACS2, can regulate membrane trafficking of multiple proteins, such as TRP, cyclic nucleotide-gated channels, vesicular monoamine transporters, and MHC-I (Piguet *et al*. 2000; Waites *et al*. 2001; Köttgen *et al.* 2005; Jenkins *et al*. 2009; Youker *et al*. 2009). Phosphorylation-mediated autoregulation of PACS proteins through the middle region (MR) also modifies their activities and subcellular site of action (Scott *et al*. 2003). PACS proteins have been shown to bind the conserved WD40 repeat-containing protein WDR37 in multiple protein interaction studies from invertebrates to mammals (Li *et al*. 2004; Malovannaya *et al*. 2011; Liu *et al*. 2020; Nair-Gill *et al*. 2021). In HEK293 cells, binding of WDR37 and PACS proteins appears to stabilize each other (Nair-Gill *et al*. 2021). However, there is very limited in vivo analysis on endogenous PACS proteins, particularly in the nervous system.

Rapid advances in sequencing and data mining have hastened the discovery of genetic variants associated with neurodevelopmental disorders, intellectual disability (ID), and autism spectrum disorder. A major challenge remains to identify their genetic etiology. Recurrent de novo missense mutations in PACS1, PACS2, and WDR37 are recently reported to be associated with syndromes characterized by a similar set of symptoms. PACS1 syndrome was first reported in 2012 when an identical de novo variant in *pacs1* (c.607C>T; p.R203W) was found in 2 unrelated children with remarkably similar ID and facial features (Schuurs-Hoeijmakers *et al*. 2012). In 2018, a PACS2 de novo mutation was reported in children with developmental and epileptic encephalopathy as well as facial dysmorphism similar to PACS1 syndrome (c.625G>A; p.E209K) (Olson *et al*. 2018). In 2019, multiple variants of WDR37 were reported in children with a spectrum of symptoms including developmental delay, ID, and epilepsy (Kanca *et al*. 2019; Reis *et al*. 2019). Emerging experimental data and structural modeling analysis suggest that several WDR37 variants appear to cause unstable proteins (Sorokina *et al*. 2021). It is unknown how PACS missense variants alter function and how all contribute to syndrome symptoms.

*Caenorhabditis elegans* has a single gene encoding PACS-1 and a single gene encoding WDR-37. In this study, we described the endogenous protein localization of PACS-1 and WDR-37 by genomic insertion of fluorescent protein tags. We examined the effects of loss of function in *pacs-1* and *wdr-37*. We edited the endogenous *C. elegans pacs-1* gene to mimic human PACS syndrome-associated variants using CRISPR/Cas9. We showed that PACS syndrome-associated variants alter PACS-1 localization. PACS-1 and WDR-37 are interdependent for expression and localization. Moreover, expression of human PACS1 can replace *C. elegans* PACS-1 function in neurons. Our work supports the evolutionary conservation of the PACS1–PACS2–WDR37 axis.

## Results and discussion

### *C. elegans pacs-1* is broadly expressed in many tissues from embryogenesis to adult

*C. elegans* PACS-1 protein shows equal similarity to both human PACS1 (33% identical/53% similar) and PACS2 (31% identical/53% similar) (Supplementary Fig. 1a). Across phylogeny, the FBR is a highly conserved region of PACS proteins (Youker *et al*. 2009). The cePACS-1 FBR is 45% identical/70% similar to hPACS-1 and 41% identical/67% similar to hPACS-2 FBRs. The residue altered in PACS1 syndrome, p.R203, lies within the FBR, corresponding to R116 in cePACS-1, and the position of the most common variant associated with PACS2 syndrome, p.E209, lies in the MR, corresponding to E205 in cePACS-1 (Supplementary Fig. 1b).

**Fig. 1.**
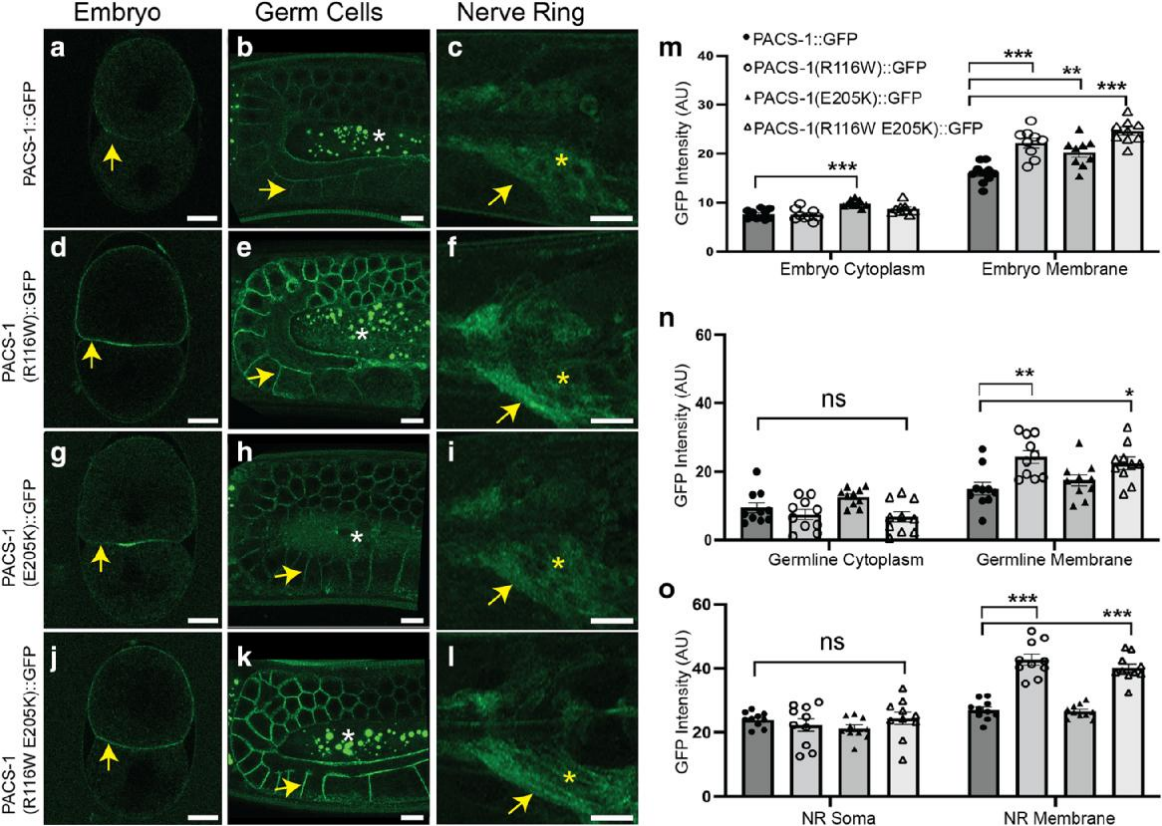
PACS1 and PACS2 disease variants alter localization of endogenously tagged *C. elegans* PACS-1 in vivo. a–c) Expression of PACS-1::GFP in 2-cell embryos a), meiotic germ cells and oocytes b), and NR c). d–f) Expression of PACS-1(R116W)::GFP in 2-cell embryos d), meiotic germ cells and oocytes e), and NR f). g–i) Expression of PACS-1(E205K)::GFP in 2-cell embryos g), meiotic germ cells and oocytes h), and NR i). j–l) Expression of PACS-1(R116W E205K)::GFP in 2-cell embryos j), meiotic germ cells and oocytes k), and NR l). In all images, embryos oriented with anterior side on top, and larvae and adults oriented with anterior end to left. Images are shown as 10-slice maximal projection for NR, 5-slice maximal projection for germ line with 0.5 μm interval, and single-slice images for embryos. Arrows point to the PACS-1::GFP; Asterisks in c), f), i), and l) point to neuronal soma in head region; Asterisks in b), e), h), and k) mark autofluorescence in germ line. Scale bars = 10 μm. m–o) Quantification of wild-type and variant PACS-1::GFP intensity in embryos m), germ cells n), and head neurons of NR o). Statistics, 1-way ANOVA with Tukey's post hoc test or Kruskal–Wallis *H* test followed by pairwise Wilcoxon rank sum exact test where *P ≤ 0.05; **P ≤ 0.01; and ***P ≤ 0.001. *n* ≥ 10 for each genotype. ns, not significant.

To determine the endogenous expression and localization of PACS-1, we used CRISPR/Cas9 genome editing technology to knock in (KI) GFP coding sequence into the *pacs-1* genomic locus, with GFP in-frame fused to the last amino acid of PACS-1. *pacs-1* is the first gene in an operon with *mdt-27* (Supplementary Fig. 1c). We verified GFP knock-in to *pacs-1* did not alter mRNA levels of *pacs-1* and *mdt-27* (Supplementary Fig. 2). *PACS-1::GFP(KI)* animals showed no overt phenotypes, including normal synaptic function tested by aldicarb sensitivity (Supplementary Fig. 3). PACS-1::GFP showed visible fluorescence in most cell types, most apparent in early embryos, germ cells, and neurons (Fig. 1a–c). This pattern was also observed in animals with mScarlet (mSc) coding sequence knocked in to the same site, though PACS-1::mSc fluorescence quenched rapidly. We therefore primarily focused on PACS-1::GFP in imaging analysis and verified key observations using PACS-1::mSc to eliminate potential bias of fluorescent proteins. PACS-1::GFP was first detected in early embryos and was enriched in the cytoplasm and near the plasma membrane of the AB cell in 2-cell embryos (Fig. 1a). Throughout the early embryo cell divisions, expression remained higher in the somatic precursor cells and lower in the early germ cell precursor cells. In late larvae and adults, PACS-1::GFP expression was apparent in both the cytoplasm and near the membrane of germ cells (Fig. 1b). In the head area, PACS-1::GFP was detectable in both the neuronal cell bodies in the ganglia and within the neuronal processes or nerve ring (NR) (Fig. 1c).

### *pacs-1* is not essential for development and growth

To determine the function of *pacs-1*, we first analyzed a partial deletion mutation, *pacs-1*(*gk325*), generated by the *C. elegans* knockout consortium, shown as *pacs-1*(partial Δ) in Supplementary Fig. 1e, which removes the MR domain and the C-terminus. *pacs-1*(partial Δ) animals showed normal body morphology and size, reproduction, and movement assayed by thrashing in liquid (Supplementary Fig. 4). Mechanosensory neuronal morphology and motor neuron synaptic number in *pacs-1*(partial Δ) were comparable to control (Supplementary Fig. 5). We assessed general synaptic transmission and stress response using aldicarb and paraquat assays and observed that *pacs-1*(partial Δ) showed normal response as control (Supplementary Fig. 3). It was previously reported that RNAi knockdown of *pacs-1* showed modest effect in the background of *dgk-1*/DGKQ diacylglycerol kinase *(lf)*, which showed hypersensitivity to aldicarb (Sieburth *et al*. 2005). We constructed double mutants of *pacs-1*(partial Δ) with *dgk-1(lf)* but found that *pacs-1*(partial Δ) did not significantly modified aldicarb sensitivity (Supplementary Fig. 3). During the process of gene editing described later, we obtained another *pacs-1* allele, designated as *pacs-1*(Δ) (Supplementary Fig. 1e), which had a premature stop after amino acid 29 as the result of a small deletion in the 5′ region of the gene and expressed greatly reduced *pacs-1* mRNA (Supplementary Fig. 2). *pacs-1*(Δ) homozygous animals resembled *pacs-1(partial Δ)* in reproduction and gross appearance of body shape and movement (Supplementary Fig. 4). Together, these observations indicate that despite early expression in embryos, *pacs-1* does not have essential roles in overall development.

### *pacs-1* with human disease variants is grossly normal in development and growth

To examine the impact of the PACS1 human disease variant in a whole animal model, we next edited *C. elegans pacs-1* genomic DNA to generate animals expressing PACS-1(R116W) (Supplementary Fig. 1f). *pacs-1(R116W)* mRNAs were expressed at the similar level as control (Supplementary Fig. 2). *pacs-1(R116W)* animals showed no discernable differences in gross phenotypes such as body morphology and size, reproduction, or movement (Supplementary Fig. 4). Mechanosensory neuronal morphology and motor neuron synapses showed overall normal pattern in *pacs-1(R116W)* (Supplementary Fig. 5). By aldicarb assay, *pacs-1*(R116W) showed normal response and did not significantly modified aldicarb hypersensitivity of *dgk-1(lf)* (Supplementary Fig. 3). As seizure is widely observed in human PACS1 patients, we also tested whether *pacs-1* mutants might show genetic interaction with *acr-2(gf)*, which exhibits seizure like behaviors, due to hyperactivation of an acetylcholine channel in the cholinergic motor neurons (Jospin *et al*. 2009). We constructed double mutants of *acr-2(gf)* with *pacs-1*(R116W) or *pacs-1*(partial Δ) and observed these double mutants grossly resembled *acr-2(gf)*. In aldicarb assay, *acr-2(gf)* showed hypersensitivity, which was not affected by either *pacs-1*(R116W) or *pacs-1*(partial Δ) (Supplementary Fig. 3).

PACS1 protein was first identified by its yeast 2-hybrid interaction with a pseudophosphorylated region of the Furin endoprotease (Wan *et al*. 1998). *C. elegans* KPC-1 and a related endoprotease, BLI-4, bear significant sequence similarity to the human Furin endoprotease, although the C-terminal regions (CTRs) do not align with the PACS1-interacting acidic cluster defined in human Furin (Q _768_ EECPSDSEEDEGRG) (Wan *et al*. 1998). Loss-of-function mutations in *kpc-1* cause a dramatic loss of PVD sensory neuron dendrites (Salzberg *et al*. 2014) (Supplementary Fig. 6). We found that neither *pacs-1*(partial Δ) nor *pacs-1*(R116W) significantly changed the dendritic structure of PVD neurons (Supplementary Fig. 6). We further constructed double mutants of *kpc-1* and *pacs-1* and found that these animals displayed a loss of PVD dendritic arborization to a similar degree as *kpc-1* single mutants (Supplementary Fig. 6). Given the high degree of conservation of the FBR, PACS proteins likely have conserved functions other than binding Furin.

### PACS-1 containing human PACS1 and PACS2 syndrome variants display increased accumulation near cell membrane

We next asked whether human PACS1 and PACS2 syndrome variants might affect the expression or localization of *C. elegans* PACS-1 protein. We edited the variant amino acid position codons in PACS-1::GFP(KI) by CRISPR/Cas9 (Supplementary Fig. 1b and f), with cePACS-1(R116W) corresponding to human PACS1(R203W) and cePACS-1(E205K) corresponding to human PACS2(E209K). The overall fluorescence pattern of PACS-1(R116W)::GFP and PACS-1(E205K)::GFP was similar to wild-type PACS-1::GFP in the cytoplasm of early embryos, oocytes, and head neurons. Most noticeably, PACS-1(R116W)::GFP showed increased intensity in membrane surrounding early embryonic cells (Fig. 1d and m), meiotic germ cells, and oocytes (Fig. 1e and n), as well as the neuronal processes of the NR (Fig. 1f and o). PACS-1(E205K)::GFP appeared slightly more enriched at the membrane of early embryonic cells (Fig. 1g and m), but not statistically significantly enriched at membrane surrounding oocytes (Fig. 1h and n) or in neuronal processes of the NR (Fig. 1i and o). We also tested how the PACS1 and PACS2 disease variants together affected PACS-1 protein expression. We made E205K as a second edit in animals expressing PACS-1(R116W)::GFP by CRISPR/Cas9 to generate PACS-1(R116W E205K)::GFP within the same protein (Supplementary Fig. 1f). PACS-1(R116W E205K)::GFP was enriched at membrane surrounding early embryonic cells (Fig. 1j and m) and germ cells (Fig. 1k and n) and in neuronal processes of the NR (Fig. 1l and o) to a similar extent as PACS-1(R116W)::GFP alone (Fig. 1m–o). Thus, introducing the PACS2 variant in combination does not appear to change the effect of the PACS1 variant on localization. It remains to be determined whether the differences between the levels of membrane enrichment of PACS-1(R116W)::GFP and PACS-1(E205K) reflect different impacts on protein function or different levels of impact on the same function.

### FBR domain of PACS-1 is important for protein stability

**Fig. 2.**
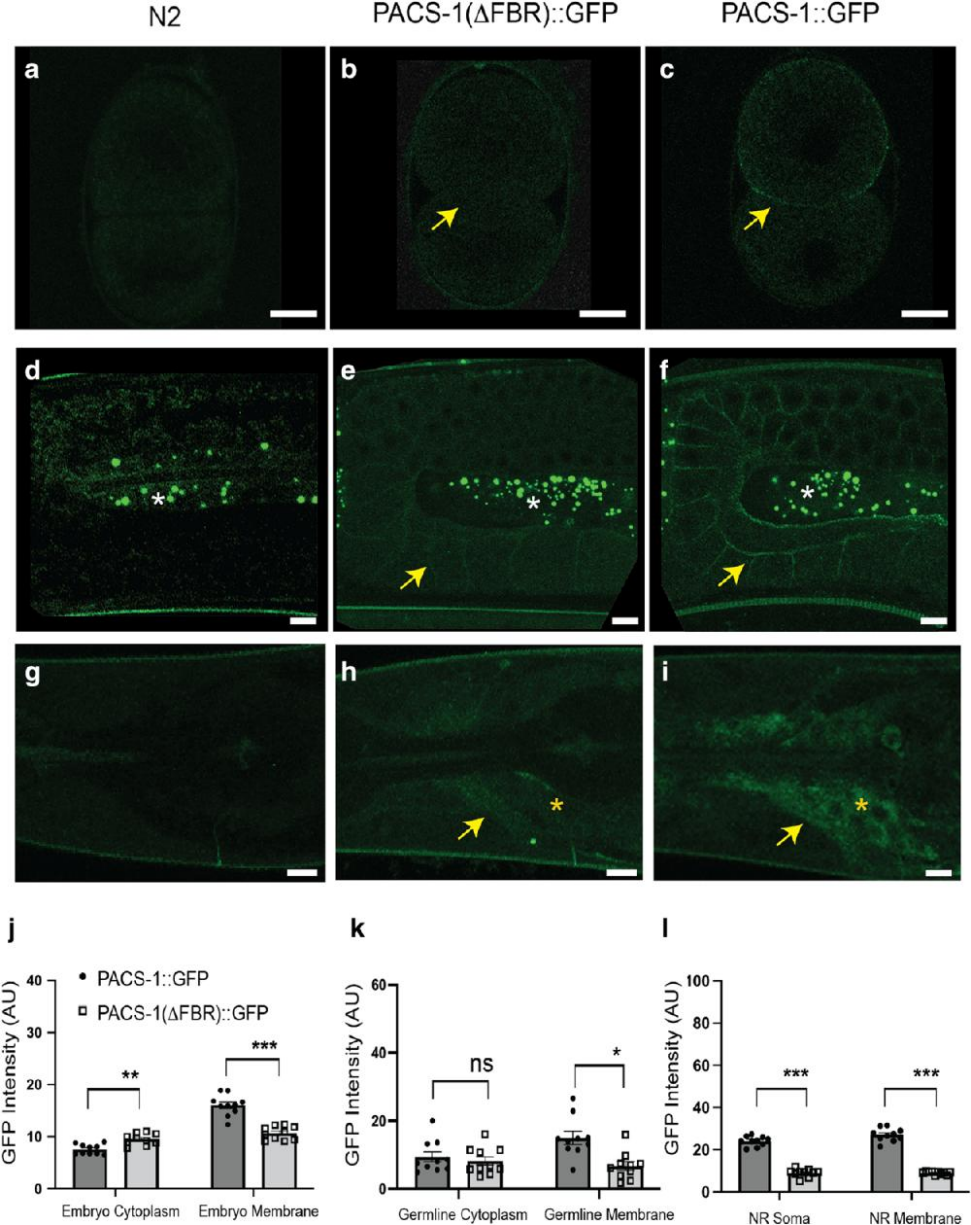
PACS-1 FBR domain is important for its expression. Shown are confocal images of 2-cell embryos a–c), germ lines d–f), and head region around the NR g–i), with genotypes indicated above. Image processing is the same as in Fig. 1. Arrows in b), c), e), f), h) and i) point to GFP signals from tagged PACS-1. Asterisks in e) and f) mark autofluorescence in germ line. Asterisks in h) and i) point to the neuronal soma in head region. Scale bars = 10 μm. j–l) Quantification of PACS-1::GFP and PACS-1(ΔFBR)::GFP expression in embryos d), germ cells e), and head neurons of the NR f). Statistics, 1-way ANOVA with Tukey's post hoc test or Kruskal–Wallis *H* test followed by pairwise Wilcoxon rank sum exact test where *P ≤ 0.05; **P ≤ 0.01; ***P≤0.001; and ns = not significant. *n* ≥ 10 for each genotype.

Since PACS1 and PACS2 disease-associated variants alter PACS-1 protein localization, we next sought to determine which domains are required for PACS-1 localization by generating endogenous in-frame deletions in PACS-1::GFP using CRISPR/Cas9. As mentioned above, *pacs-1* is the first gene in an operon with *mdt-27* (Supplementary Fig. 1c), which encodes a protein of the mediator family. Unexpectedly, we found that any genome editing that resulted in deletions in the 3′ region of *pacs-1*, corresponding to the coding sequences for the MR and CTR of PACS-1, led to reduced expression of *mdt-27* mRNA. This observation may likely reflect genomic changes to the operon structure and confounds meaningful interpretation of observed changes in PACS-1 expression levels and localization patterns. Nonetheless, we were able to obtain an in-frame deletion of the FBR, denoted as *pacs-1*(ΔFBR)::*gfp* (Supplementary Fig. 1e). We also verified that in these animals, mRNA levels of *pacs-1* and *mdt-27* were not altered (Supplementary Fig. 2). While we could detect PACS-1(ΔFBR)::GFP in all tissues that expressed PACS-1::GFP, we found that PACS-1(ΔFBR)::GFP mutants showed significantly reduced levels of fluorescence in both cytoplasm and membrane in early embryos (Fig. 2b), germ cells (Fig. 2e), and head neurons (Fig. 2h). This observation suggests that the FBR is likely important for the stability or turnover of the PACS-1 protein.

### *wdr-37* is required for stable expression of PACS-1

**Fig. 3.**
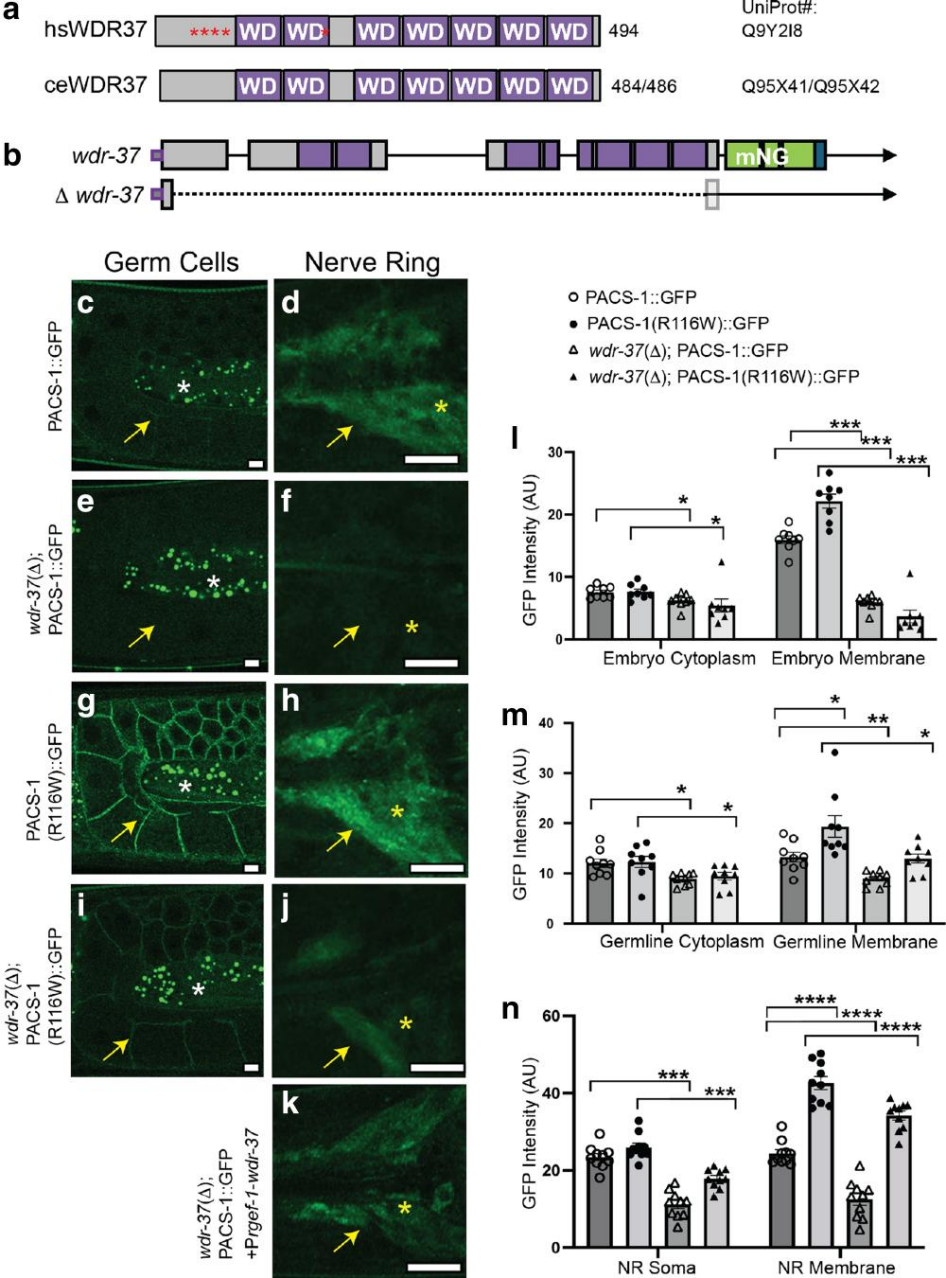
WDR-37 is important for PACS-1 localization in soma. a) Schematic of human WDR37 and *C. elegans* WDR-37 proteins showing WD domain repeats and human variant positions (asterisks). *C. elegans wdr-37* is predicted to generate 2 isoforms (UniProt Q95X41 and Q95X42) due to the use of an alternative splice site at the 3′ end of the first intron. However, in this study, *wdr-37* cDNA matched the shorter isoform UniProt Q95X41. b) Illustration of *wdr-37* gene structure showing sites of CRISPR-generated genomic insertion of mNG with Flag tag and deletion *wdr-37*(Δ)(*ju1847*). c and d) PACS-1::GFP in germ cells c) and head neurons of the NR d). e and f) PACS-1::GFP in germ cells e) and head neurons f) of animals with *wdr-37* deletion [*wdr-37*(Δ)]. g and h) Variant PACS-1(R116W)::GFP in germ cells g) and head neurons of the NR h). i and j) Variant PACS-1(R116W)::GFP in germ cells i) and head neurons j) of animals with *wdr-37*(Δ). Shown were confocal images of 10-slice maximal projection with 0.5 μm interval; Arrows point to GFP signals; Asterisks in d), f), h), j) and k) mark neuronal soma in the head. Asterisks in c), e), g) and i) mark autofluorescence in the germ line. Three independent transgenic lines were scored visually under compound fluorescence microscope. Scale bars = 10 μm. k) Expression of PACS-1::GFP in head neurons (yellow arrowhead and asterisk) in *wdr-37*(Δ) animals was rescued with transgenic expression of *Prgef-1*-*wdr-37* cDNA. In *wdr-37*(Δ) animals, nontransgenic animals showed no to very faint PACS-1::GFP fluorescence, whereas transgene-carrying animals (*n* > 15) all showed readily visible fluorescence. l–n) Quantification of PACS-1::GFP and PACS-1(R116W)::GFP expression with and without *wdr-37*(Δ) in embryos l), germ cells m), and head neurons of the NR n). Statistics, 1-way ANOVA with Tukey's post hoc test where *P ≤ 0.05; **P ≤ 0.01; ***P ≤ 0.001; ****P ≤ 0.0001. *n* ≥ 10 for each genotype.

The WD domain-containing protein, WDR37/WDR-37, is a conserved interacting partner of PACS1/PACS-1 and PACS2 in human, mouse, and *C. elegans* (Li *et al*. 2004; Nair-Gill *et al*. 2021; Sorokina *et al*. 2021). *C. elegans* WDR-37 (C05D2.10a) and human WDR37 share 43% identity/59% sequence similarity (Fig. 3a). To determine the function of *wdr-37*, we generated deletion alleles of *wdr-37*, designated *wdr-37*(Δ), by CRISPR/Cas9 (Fig. 3b). *wdr-37*(Δ) animals had normal gross morphology and body size, reproduction, and movement (Supplementary Fig. 4). However, these animals showed a dramatic reduction of PACS-1::GFP in embryos (Fig. 3l), germ cells (Fig. 3e and m), and neurons (Fig. 3f and n). We transgenically expressed wild-type *wdr-37* cDNA using a panneural promotor (*Prgef-1*) in *wdr-37*(Δ) and observed that the expression levels of PACS-1::GFP were restored to normal (Fig. 3k). Since *wdr-37*(Δ) animals had comparable levels of *pacs-1* mRNA (Supplementary Fig. 2), we interpret that the reduced PACS-1::GFP expression in *wdr-37*(Δ) may indicate a dependence on WDR-37 for PACS-1 protein stability or turnover. We also tested whether *wdr-37* might affect human variant PACS-1(R116W)::GFP. By confocal imaging analysis, we found that *wdr-37*(Δ) animals showed a significant reduction in overall levels of PACS-1(R116W)::GFP in embryos (Fig. 3l), germ cells (Fig. 3i and m), and neurons (Fig. 3j and n), although PACS-1(R116W)::GFP remained visible at the membranous regions between germ cells (Fig. 3i and m) and the membranous NR processes (Fig. 3j and n). Therefore, *wdr-37* appears to be generally required for the stability of PACS-1 protein.

### WDR-37 colocalizes with PACS-1 and *pacs-1* regulates WDR-37 expression

**Fig. 4.**
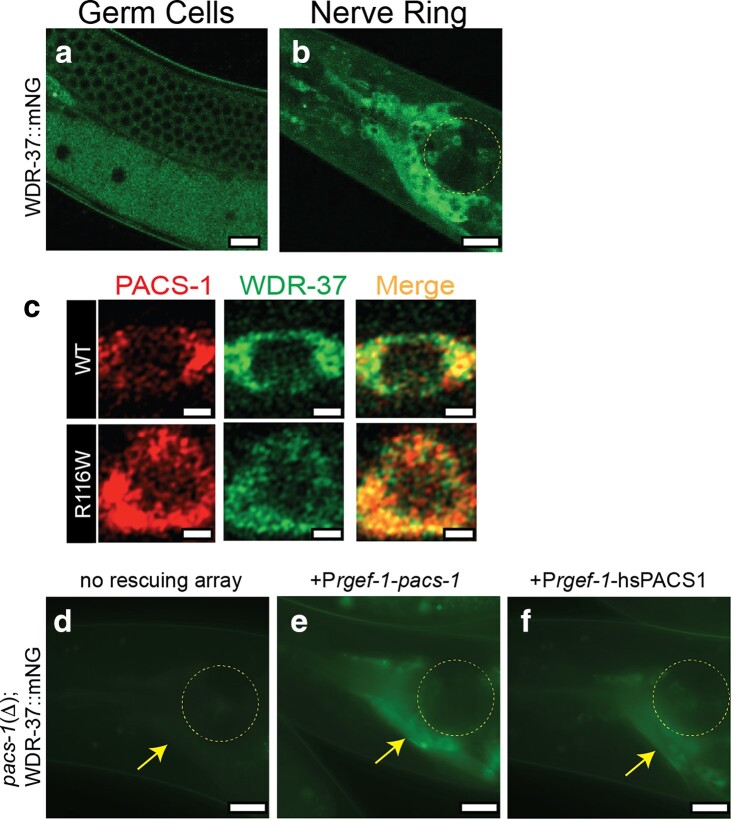
WDR-37 localization overlaps with and depends on PACS-1, and human PACS1 in neurons can complement the function of *Ce*PACS-1. a and b) WDR-37::mNG (*syb5283*) expression in meiotic germ cells and oocytes a) and in head neurons b). Scale bars = 10 μm. c) Airyscan images showing that PACS-1::mSc and variant PACS-1(R116W)::mSc overlap with WDR-37::mNG. Scale bars = 1 μm in c). d–f) Expression of WDR-37::mNG in head neurons shown by arrows in *pacs-1*(Δ) animals d) and with extrachromosomal arrays expressing *C. elegans pacs-1* cDNA (*Prgef-1*-*pacs-1* cDNA) e) and human PACS1 cDNA (*Prgef-1*-hsPACS1 cDNA) f) in neurons. Images were taken using Zeiss Axio Imager M2 compound microscope under identical conditions. Scale bars = 10 μm. Adult animals oriented with anterior end to left. Four independent transgenic lines expressing P*_rgef-1_*-*pacs-1* cDNA and 10 independent transgenic lines expressing P*_rgef-1_*-hsPACS1 cDNA were scored. In *pacs-1*(Δ) animals, WDR-37::mNG showed very faint fluorescence, whereas transgene-carrying animals expressing either *C. elegans pacs-1* cDNA or human PACS1 cDNA all showed visibly detectable fluorescence in all lines scored.

To further examine how PACS-1 and WDR-37 might function together in *C. elegans*, we generated a C-terminal mNeonGreen (mNG)-tagged WDR-37 by CRISPR/Cas9 genomic insertion of mNG coding sequence in the endogenous *wdr-37* locus (Fig. 3b). We found that WDR-37::mNG was expressed in a similar pattern as PACS-1::GFP, with most prominent expression in germ cells (Fig. 4a) and neurons (Fig. 4b). However, in germ cells, WDR-37::mNG was more prominent in the cytoplasm and not enriched at the surrounding membrane (Fig. 4a). Within ventral motor neurons, PACS-1::mSc and WDR-37::mNG largely colocalized (Fig. 4c, top). PACS1 disease variant, PACS-1(R116W)::mSc, retained colocalization with WDR-37::mNG (Fig. 4c, bottom), suggesting that the PACS1 disease variant may not alter its physical interaction with WDR37.

Murine PACS1 and WDR37, when expressed in HEK cells, are mutually required for stability (Nair-Gill *et al*. 2021); we tested whether *pacs-1* was required for expression of WDR-37::mNG and found that WDR-37::mNG levels were reduced in neurons of *pacs-1*(Δ) animals (Fig. 4d). Expression of *C. elegans pacs-1* cDNA in neurons restored neuronal expression of WDR-37::mNG in *pacs-1*(Δ) animals (Fig. 4e), supporting a conclusion that PACS-1 functions cell-autonomously to promote neuronal expression of WDR-37.

### Expression of human PACS1 can complement *C. elegans* PACS-1 function in neurons

To address whether human PACS1 and *C. elegans* PACS-1 behave as functional orthologs, we expressed human PACS1 cDNA in *C. elegans* neurons and assessed whether it could also rescue the reduced levels of WDR-37::mNG observed in *pacs-1*(Δ). We found that expression of human PACS1 cDNA in *C. elegans* neurons, using a pan-neuronal promoter *prgef-1*, rescued the neuronal expression of WDR-37::mNG (Fig. 4f). This analysis shows that human PACS1 cell-autonomously supports stability or expression of *C. elegans* WDR-37/WDR37, similar to *cePACS-1*, further supporting *C. elegans* as a relevant model for understanding conserved function and identification of regulatory network components for the PACS1/PACS2/WDR37 axis.

### Conclusion

Pacs1/2 and Wdr37 are recently identified rare disease-associated genes. In this study, we have reported the endogenous protein expression of PACS-1 and WDR-37 in living *C. elegans* and analyzed genetic null or strong loss of functions in each gene. We find that cePACS-1 and ceWDR-37 largely colocalize in the somatic cytoplasm of many tissues and are mutually required for expression, providing in vivo evidence for the previous biochemical findings for mammalian PACS1 and WDR37 in cultured cells (Nair-Gill *et al*. 2021). However, animals lacking *pacs-1* or *wdr-37* display normal development from embryo to adults. We have not detected discernable abnormal features in neuronal morphology, synapse development, and neuron functions in multiple types of neurons tested. These data suggest that the 2 genes may act in parallel to other factors in specific cellular processes. Through editing endogenous genes, we further characterized the effects of PACS1 and PACS2 syndromic variants and found that the human PACS1 and PACS2 variants in cePACS-1 altered protein localization. As studies of mammalian PACS proteins have revealed their broad roles in sorting and retrieval of multiple proteins, we speculate that PACS syndromic-associated proteins may disrupt cellular function by altering the subcellular distribution of a cohort or a select set of proteins depending on the types of cells. Lastly, we demonstrated that both *C. elegans* PACS-1 and human PACS1 can functionally complement PACS-1 in neurons. Our findings reveal the effects of human variants in a genetically amenable invertebrate model providing potential strategies to identify regulatory network components that may contribute to understanding the molecular underpinnings of PACS/WDR37 syndromes.

## Materials and methods

### *C. elegans* genetics

Wild-type *C. elegans* is the N2 Bristol variant (Brenner 1974). Strains were maintained under standard conditions on nematode growth media seeded with *Escherichia coli* OP50 as described (Brenner 1974) and listed in Supplementary Table 1. Transgenes and expression constructs are listed in Supplementary Table 3.

### CRISPR/Cas9 genome editing

Guide RNAs for editing *pacs-1* and deletions of *pacs-1* and *wdr-37* were selected using the CRISPR guide RNA selection tool (http://genome.sfu.ca/crispr/) and Integrated DNA Technologies crRNA design tools and listed in Supplementary Table 2. *pacs-1::gfp* and *pacs-1::mSc* knock-ins were made using SunyBiotech (Fuzhou, China). Human PACS variant alleles and *wdr-37*(*ju1847*) deletion were generated using crRNA and repair oligos along with purified Cas9, tracrRNA, *unc-58* crRNA, and *unc-58*(*gf*) repair oligo as described (Arribere *et al.* 2014). *pacs-1* deletion alleles were generated similarly, except using *dpy-10* crRNA as described (Paix *et al.* 2015). Edited mutations were confirmed by Sanger sequencing (Eton Bioscience, Inc.) and were outcrossed at least twice with N2. Genotyping primers are listed in Supplementary Table 2.

### Fluorescence microscopy

Animals were generally immobilized in a drop of M9 solution with 1 mM levamisole on a 4% agar pad or 10% agarose pad. Most fluorescence images were collected with a 63× oil immersion objective using a Zeiss LSM800 confocal microscope. Representative head neurons/NR images are shown in 10-slice *z*-stack maximum projection at 0.5 μm interval centered on middle of pharynx prepared using Fiji (ImageJ). Germ line images are presented as middle 5-slice *z*-stack maximum projection at 0.5 μm interval. All embryo images are shown as a single slice where the nucleus shows the biggest area. Cytoplasm and membrane regions were quantified by averaging the mean gray value from 3 regions of interest. Images of ventral cord neurons expressing both WDR-37::mNG and PACS-1::mSc were collected with a 63× oil immersion objective using a Zeiss LSM900 Airyscan microscope. For *pacs-1(ju2014)* rescue experiments, images of head neurons expressing WDR-37::mNG were collected with a 63× oil immersion objective on a Zeiss Axio Imager M2 compound microscope under identical settings.

### Statistical analysis

Statistical analysis was performed using GraphPad Prism 9 or 10 (GraphPad Software, Inc.) and using RStudio (R Studio Team 2020). The statistical significance was determined using 1-way ANOVA or 2-way ANOVA with Tukey's post hoc test, nonparametric Kruskal–Wallis *H* test followed by pairwise Wilcoxon rank sum exact test, or unpaired, 2-tailed *t*-tests. A *P* > 0.05 was considered not significant. Fisher's exact test was used for the neuron morphology assessment. *P* < 0.05 (*); *P* < 0.01 (**); *P* < 0.001 (***); and *P* < 0.0001 (****) were considered significant differences. Data are represented as mean ± SEM.

## Supplementary Material

iyae118_Supplementary_Data

## Data Availability

Strains and plasmids are available upon request. The authors affirm that all data necessary for confirming the conclusions of the article are present within the article, figures, and tables. Supplemental material available at GENETICS online.
