# Supplementary material for: PACS-1 variant protein is aberrantly localized in Caenorhabditis elegans model of PACS1/PACS2 syndromes
Source: Genetics. 2024 Jul 20;228(2):iyae118. doi: 10.1093/genetics/iyae118 (PMC11457933; doi:10.1093/genetics/iyae118)
Supplement: iyae118_Supplementary_Data [file iyae118_supplementary_data.pdf]

Supplemental Materials

PACS-1 variant protein is aberrantly localized in *C. elegans* model of PACS1/PACS2 syndromes

Dana T. Byrd, Ziyuan Christina Han, Christopher A. Piggott, Yishi Jin

Supplemental materials and methods

Figure S1: Illustration of PACS-1 homology with human PACS1 and PACS2, and *pacs-1* genetic mutants.

Figure S2: RT-PCR analysis of *pacs-1* and *mdt-27* mRNAs in various *pacs-1* and *wdr-37* mutants.

Figure S3: Aldicarb and paraquat assays for various *pacs-1* strains.

Figure S4: Body morphology, body length, reproduction and movement in various *pacs-1* and *wdr-37* mutants.

Figure S5: Analysis of mechanosensory neuron morphology and motor neuron synaptic patterns in various *pacs-1* mutants.

Figure S6: Analysis of PVD dendritic morphology in various *pacs-1* mutants.

Table S1: Strains used in this study
Table S2: crRNA and oligo sequences
Table S3: DNA expression constructs

Supplemental Materials and methods:

Body length assessment

To image and quantify body length, at least 10 L4 animals were mounted on 4% agar pad and anesthetized with 1mM levamisole. Images were taken using Zeiss Axio Imager A2 compound scope at 10× magnification under identical DIC settings. The body length for each animal was analyzed by drawing a segmented line from head to tail using Fiji (ImageJ). Animals were maintained at 20°C and the genotypes were blinded until the data analysis was completed.

Reproduction assessment

Five L4 hermaphrodite animals for each genotype were placed on seeded individual NGM plates and transferred to new plates every 24 hours for 5 consecutive days at 20°C. The total number of hatched animals and unhatched embryos were counted by removing all animals every day for each plate, with the genotypes blinded until the data collection was completed.

Thrashing assay

Individual L4 animals were placed in a drop of M9 on a microscope slide with 4% agar pad and climatized for 30 seconds to 1 minute before video recording. A single thrash was defined as the large movement of the head or tail back and forth. The number of thrashes performed by the animal in 1 minute was counted.

Neuron morphology assessment

At least 20 L4 animals expressing *muIs32 (Pmec-7-GFP)* were placed on seeded NGM plates and maintained for 24 hours at 20°C to reach day-1 adults. Animals were mounted on 4% agar pad and anesthetized by 1mM levamisole. The morphology of anterior lateral mechanosensory (ALM) neurons were scored under Zeiss Axio Imager A2 compound scope under GFP channel at 40× magnification with genotypes blinded to the observer. Ectopic posterior neurite outgrowth phenotype was scored as the neurite grew from the soma was longer than the diameter of the soma. At least 3 observations were conducted under the same condition.

Density of *en passant* synapses of the GABAergic motor neurons was assessed using *juIs1* (P*unc-25*-SNB-1::GFP) marker. Day-1 adults were mounted on 4% agar slides in M9 with 1mM levamisole for imaging on a Zeiss Axio Imager A2 compound microscope with 40x objective. Images of the ventral nerve cord and dorsal nerve cord between VD10 and VD11 were each captured from at least 4 animals under identical conditions. Number of discrete GFP puncta were

manually counted from each image, genotype blind, and normalized to number puncta per 100μm using Fiji(ImageJ).

wyIs378 (*ser-2prom-3p*::*myr*-GFP) was used to assess PVD neuron morphology. Late L4 animals were mounted on 4% agar slides in M9 with 1mM levamisole for imaging on a Zeiss Axio Imager A2 compound microscope with 40x objective. For each genotype, 4-6 animals were selected for imaging based on their "Christmas tree" vulval morphology to verify all animals were at the same developmental stage. Three focal planes were captured for each animal under identical conditions to collect an in-focus view of the primary, mid-secondary, and tertiary PVD processes just anterior of the PVD cell bodies. The 3 focal planes were then stacked into a single image using Fiji (ImageJ). Number of secondary and quaternary processes were counted manually and normalized to number processes per 100μm.

Molecular biology and transgenesis:

For RT-PCR, mixed stage worms were cultured under the same standard conditions. Total RNA was isolated using either TRIzol (Thermo Fisher Scientific) or RNeasy Plus Universal Mini Kit (Qiagen cat. no. 73404). All RNA samples were repurified using the TURBO DNA-free kit (Thermo Fisher Scientific) or iScript ™ gDNA Clear cDNA Synthesis Kit (BIO-RAD cat. no 1725034). Reverse transcription was made using around 1µg of total RNA and the Superscript III RT kit (Thermo Fisher Scientific) or iScript ™ gDNA Clear cDNA Synthesis Kit (BIO-RAD cat. no 1725034). To qualitatively assess the abundance of cDNA, all the cDNA samples were diluted into 200ng/μL and were amplified for 30x cycles using Primers SD20344 and SD20346 that target exon 6 and exon 8, respectively. SD20343 and SD20345 were used to confirm the sequence of cDNAs (Table S2). *mdt-27* in each cDNA sample was amplified using SD20382 and SD20383 (Table S2). *act-1* control was amplified from each sample using SD20368 and SD20369 (Table S2).

For *C. elegans* expression constructs, *pacs-1* and *wdr-37* cDNAs were amplified from an N2 cDNA library (PIGGOTT *et al.* 2021) with SD20414 and SD20415 for *pacs-1* or SD20416 and SD20417 for *wdr-37* (Table S2) using Phusion HF DNA Polymerase (NEB) and cloned into pCR8 by TOPO-TA cloning (Thermo Fisher Scientific). After verifying the sequences, *pacs-1* and *wdr-37* cDNAs were then recombined into Gateway plasmids containing the pan-neuronal promoter P*rgef-1* (pCZGY51 and pCZGY66) using Gateway LR Clonase II (Thermo Fisher Scientific), generating pCZGY3630 and pCZGY3615, respectively (Table S3). Human PACS1 cDNA

(pENTR_PACS1_HA3; Gleeson Lab) was recombined into destination plasmid containing the pan-neuronal promoter P*rgef-1* (pCZGY66) using Gateway LR Clonase II (Thermo Fisher Scientific), generating pCZGY3631 (Table S3).

Transgenic lines were made by microinjection as previously described (MELLO *et al.* 1991). Plasmids and coinjection markers together with injection concentrations are listed in Table S3.

Aldicarb sensitivity assay:
30 day-1 adult animals were transferred to fresh plates containing 0.5 mM or 1 mM aldicarb. Animals were scored for paralysis every 15 or 30 min by gently touching the animal with a platinum wire. Final sample size for each assay was 20-25 animals due to some animals crawling off the plate.

Paraquat sensitivity assay:
To prepare paraquat plates, 18 µL of 1 M paraquat were mixed with 300 µL ddH_2O and spread over seeded 5 cm NGM plates and air dried 3 hours at room temperature. 20 day-1 adults were placed on each plate, incubated at 20°C, and monitored daily for survival.

Figure S1

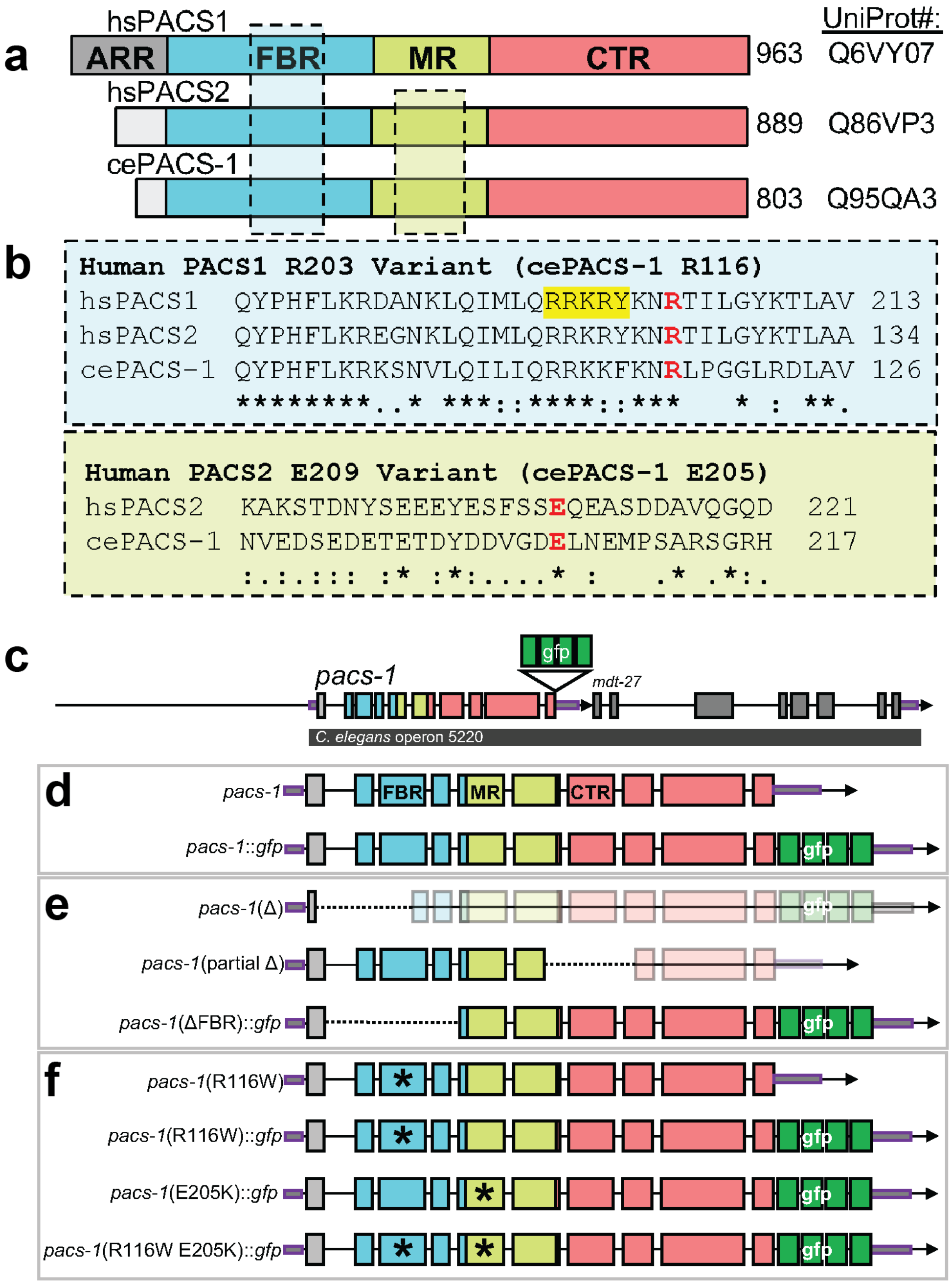

Figure S1: Illustration of PACS-1 homology with human PACS1 and PACS2, and *pacs-1* genetic mutants.

(a) Human PACS1, PACS2, and *C. elegans* PACS-1 protein schematics. ARR is atrophin-1-related region (gray), FBR is furin binding region (cyan), MR is middle region (green), and CTR is C-terminal region (red). Boxed regions match alignments in B. (b) Clustal Omega sequence

alignments of human disease variant regions: PACS1 R203W variant region (cyan box) and PACS2 E209K region (green box) with variant residues (red) and CK2 binding site (yellow highlight). (c) *C. elegans pacs-1* (T18H9.7) gene structure showing genomic insertion site of GFP (same site for mSc), and downstream gene within operon, *mdt-27*. (d) Wild type expected gene products for *pacs-1* without GFP (N2) and *pacs-1*::*gfp* (*pacs-1*(*syb2274*)). (e) Loss-of-function expected gene products from *pacs-1*(Δ) (*pacs-1*::*gfp*(*ju2014*)), *pacs-1*(partial Δ) (*pacs-1*(*gk325*)), and *pacs-1*(ΔFBR)::*gfp* (*pacs-1::gfp*(*ju1966*)). While *pacs-1*(Δ) was generated in the gfp KI strain, *pacs-1::gfp(syb2274),* coding region 3' of the deletion, including gfp, is out of frame in *pacs-1*(Δ). (f) Human variant expected gene products *pacs-1*(R116W) (*pacs-1*(*ju1873*)), *pacs-1*(R116W)::*gfp* (*pacs-1::gfp* (*ju1823*)), *pacs-1*(E205K)::*gfp* (*pacs-1*::*gfp* (*ju1999*)), and *pacs-1*(R116W E205K)::*gfp* (*pacs-1*(R116W)::*gfp* (*ju2021*)).

Figure S2

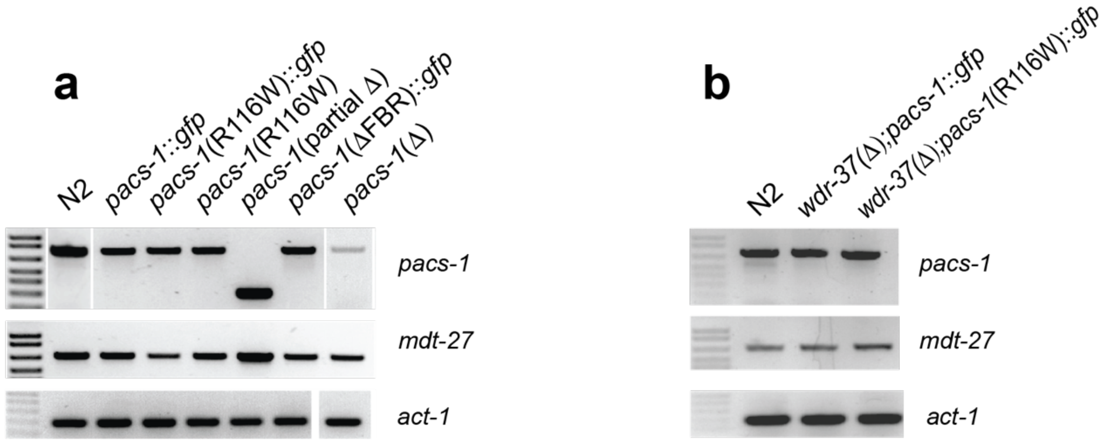

Figure S2: RT-PCR analysis of *pacs-1* and *mdt-27* mRNAs in various *pacs-1* and *wdr-37* mutants.

(a) rtPCR of *mdt-27*, *pacs-1* and *act-1* control using equivalent total cDNA from the following strains: N2, *pacs-1*::*gfp* (*syb2274*), *pacs-1*(R116W)::*gfp* (*ju1823*), *pacs-1*(R116W) (*ju1873*), *pacs-1*(partial Δ) (*gk325*), *pacs-1*(Δ FBR)::*gfp* (*ju1966*), and *pacs-1*(Δ) (*ju2014*).

(b) rtPCR of *mdt-27*, *pacs-1* and *act-1* control using equivalent total cDNA from the following strains: N2, *wdr-37(ju1847);pacs-1*::*gfp* (*syb2274*), *wdr-37(ju1847)*;*pacs-1*(R116W)::*gfp* (*ju1823*).

Figure S3

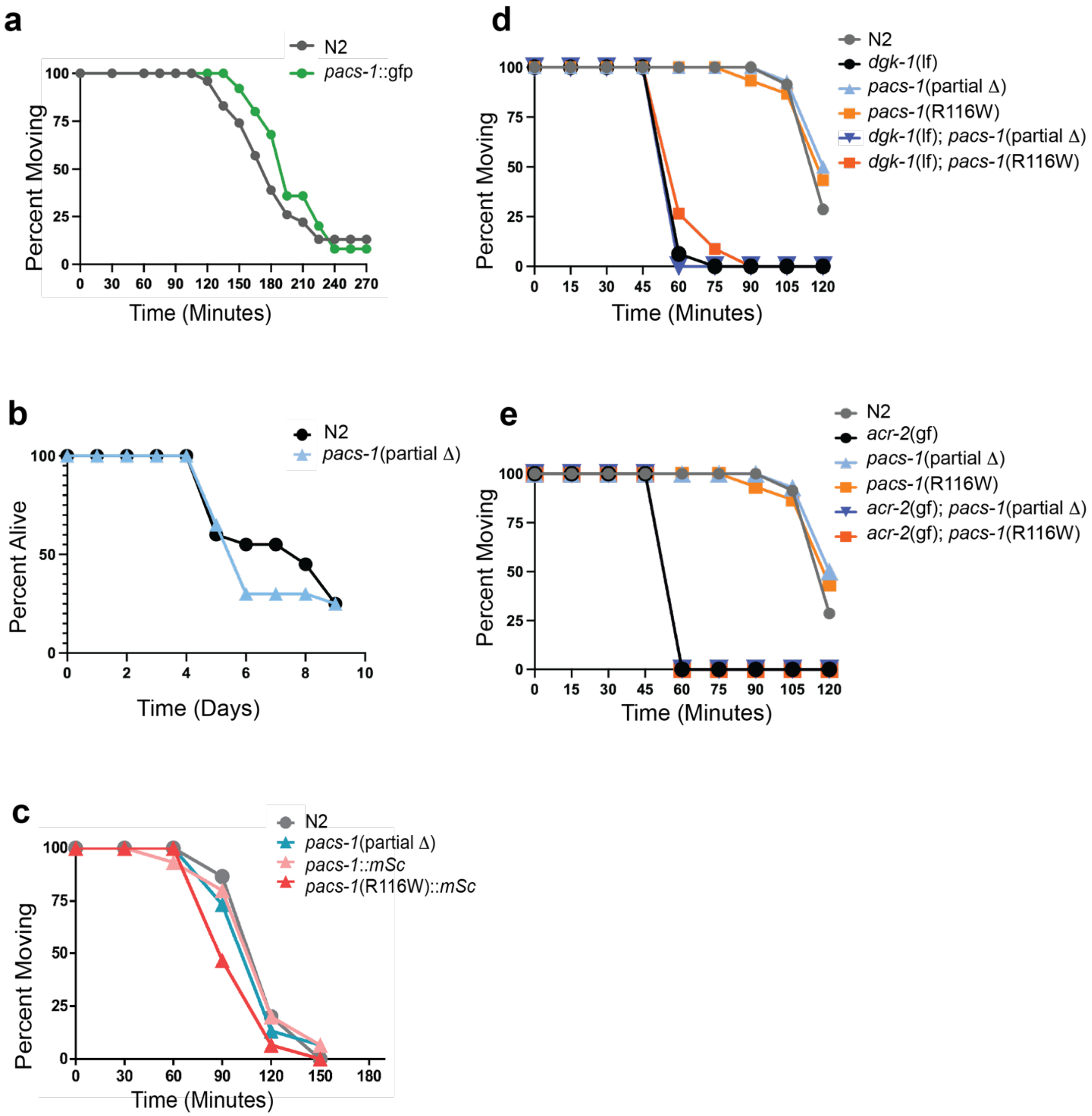

Figure S3: Aldicarb and paraquat assays for various *pacs-1* strains.

(a) Aldicarb sensitivity assay showing percentage of animals moving versus time in minutes for N2 and *pacs-1*::*gfp* (*syb2274*). GFP KI does not alter aldicarb sensitivity. Assayed on 0.5 mM

Aldicarb plates. n≥20 for each genotype. (b) Paraquat sensitivity assay showing the percent of animals alive versus time in days. *pacs-1*(partial Δ) and wild type (N2) animals have similar sensitivity to paraquat. n=20 for each group. (c) Aldicarb sensitivity assay showing percentage of animals moving versus time in minutes for N2, *pacs-1*(partial Δ) (*gk325*), *pacs-1*::*mSc* (*syb2272*), and *pacs-1*(R116W)::*mSc* (*ju1827*). PACS-1::mSC KI and *pacs-1*(partial Δ) loss of function do not alter aldicarb sensitivity. Assayed on 1 mM aldicarb plates. n≥20 for each genotype. (d) Aldicarb sensitivity assay showing the percent of animals moving versus time in minutes. *pacs-1*(partial Δ) (*gk325* light blue line) and *pacs-1*(R116W) (*ju1873* light orange line) have similar aldicarb sensitivity as wild type animals (N2 gray line) and do not significantly alter aldicarb sensitivity of *dgk-1*(*lf*) (*nu62* black line). Assayed on 0.5 mM aldicarb plates. n=20-30 for each genotype. (e) Aldicarb sensitivity assay showing the percent of animals moving versus time in minutes. *pacs-1*(partial Δ) (*gk325* blue line) and *pacs-1*(R116W) (*ju1873* orange line) do not significantly alter aldicarb sensitivity of *acr-2 (n2420gf*) (black line). Assayed on 0.5 mM aldicarb plates. n=20-30 for each genotype.

Figure S4

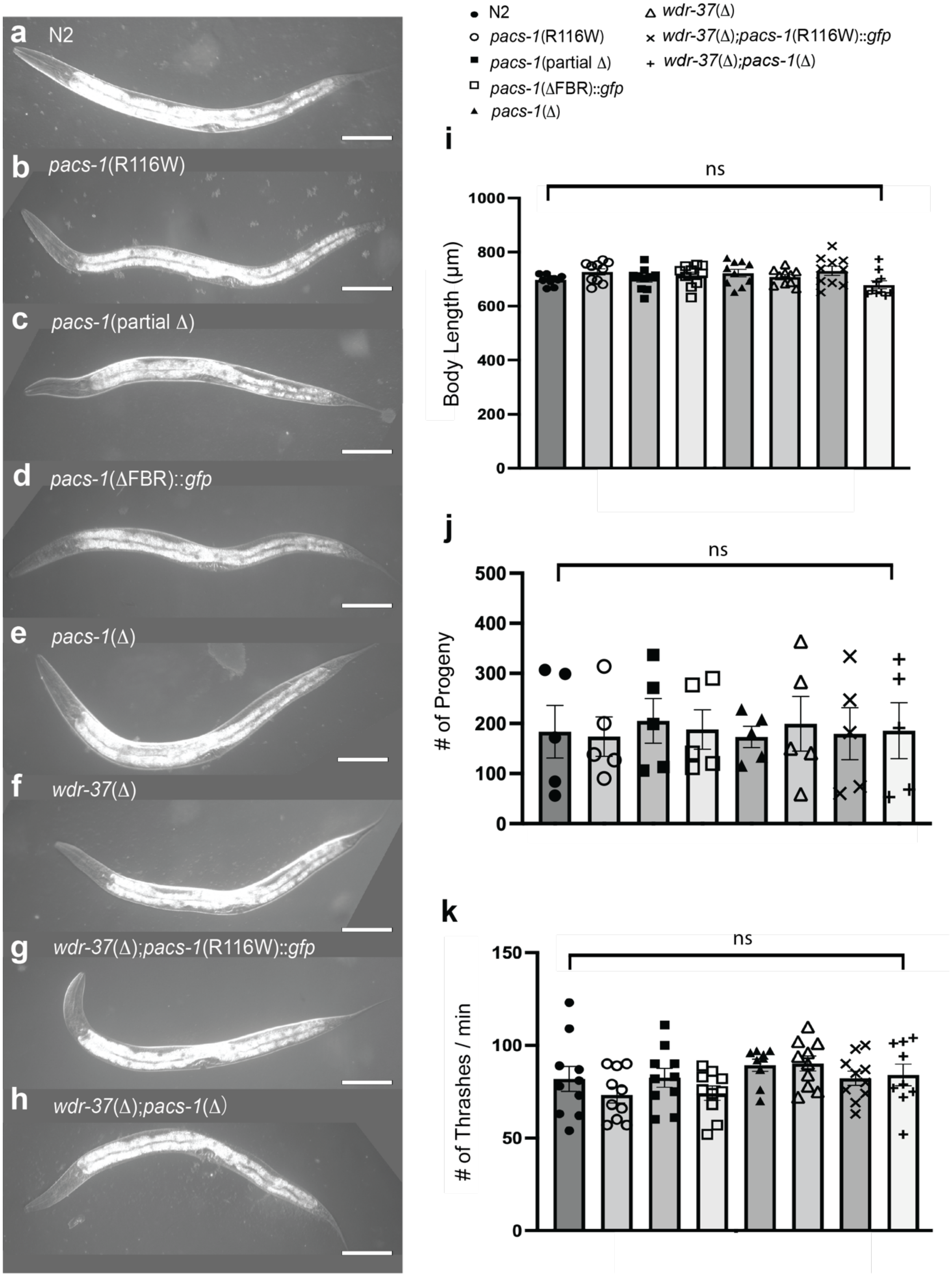

a N2
b pacs-1(R116W)
c pacs-1(partial Δ)
d pacs-1(ΔFBR)::gfp
e pacs-1(Δ)
f wdr-37(Δ)
g wdr-37(Δ);pacs-1(R116W)::gfp
h wdr-37(Δ);pacs-1(Δ)
N2
pacs-1(R116W)
pacs-1(partial Δ)
pacs-1(ΔFBR)::gfp
pacs-1(Δ)
wdr-37(Δ)
wdr-37(Δ);pacs-1(R116W)::gfp
wdr-37(Δ);pacs-1(Δ)
i
ns
Body Length (μm)
0
200
400
600
800
1000
j
ns
# of Progeny
0
100
200
300
400
500
k
ns
# of Thrashes / min
0
50
100
150

Figure S4: Body morphology, body length, reproduction and movement in various *pacs-1* and *wdr-37* mutants.

(a-h) Representative images of gross morphology from wild-type(N2) (a), *pacs-1(R116W)* (b), *pacs-1(partial Δ)* (c), *pacs-1(Δ FBR)::gfp* (d), *pacs-1(Δ)* (e), *wdr-37(Δ)* (f), *wdr-37(Δ);pacs-1(R116W)::gfp* (g), *wdr-37(Δ);pacs-1(Δ)* (h). Scale bars=100μm. (i) Quantification of body length in each group. (j) The quantification of progeny over 5 consecutive adult stages. (k) Quantification of the number of thrashes within 1 min. n = 10 for each group in i and k, n = 5 for each group in j. One-way ANOVA was used for i and k. Two-way ANOVA was used in j. ns = not significant.

Figure S5

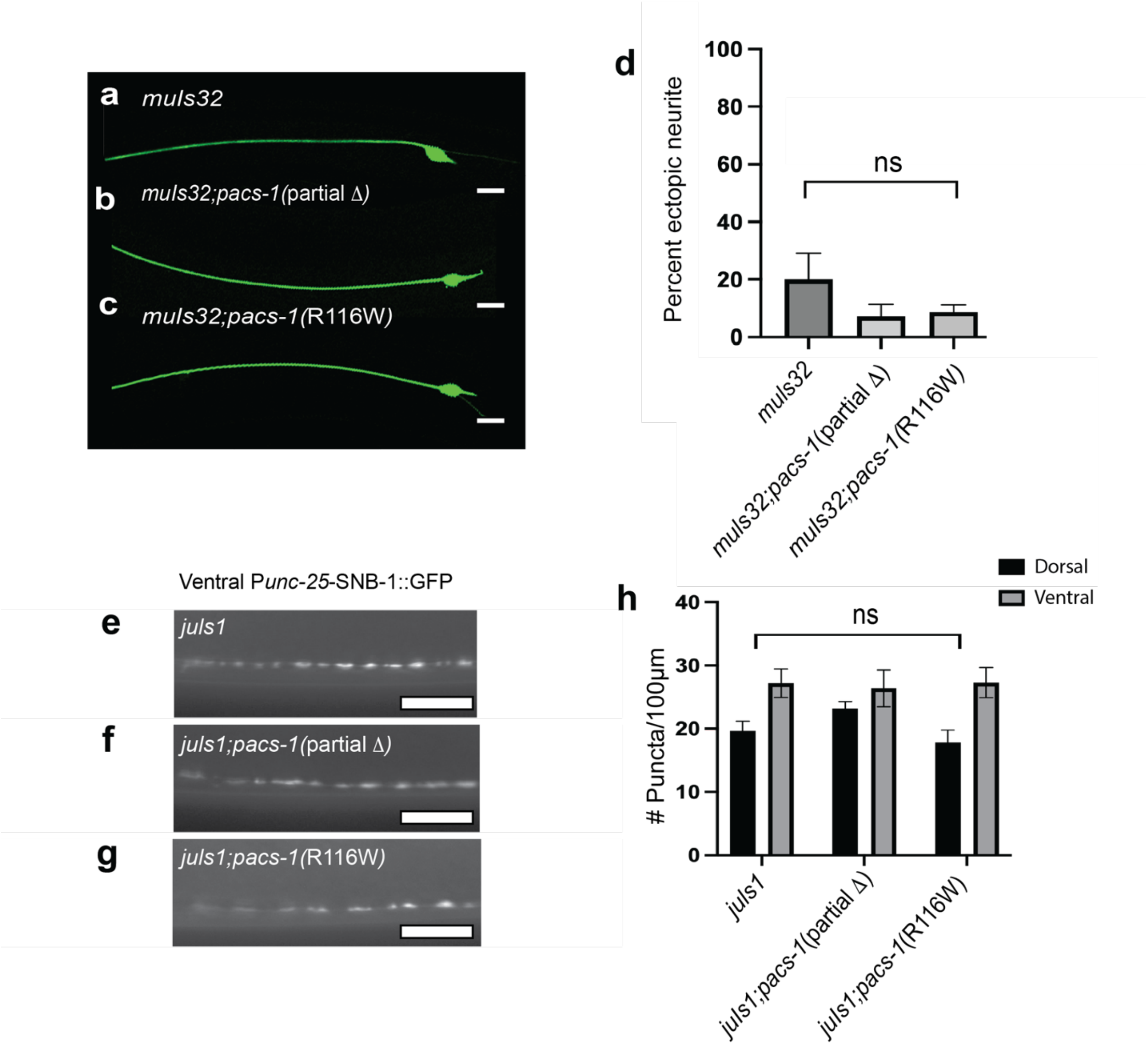

Figure S5. Analysis of mechanosensory neuron morphology and motor neuron synaptic patterns in various *pacs-1* mutants.

(a-c) Representative images of anterior lateral mechanosensory (ALM) neuron for *muIs32* (a), *muIs32;pacs-1(*partial Δ*)* (b), *muIs32;pacs-1(*R116W*)* (c). (d) Quantification of the percentage of ectopic neurite outgrowth from the ALM soma. An ectopic neurite is defined as a neurite that is longer than the soma diameter. 3 independent observations (n=3) were conducted on at least 20 animals in each group each time. Fisher's exact test was used for f. ns= not significant. (e-g) Representative images of the synaptic vesicle associated protein marker, P_{unc-25}-SNB-1::GFP (*juIs1*) in WT (e), *pacs-1*(partial Δ) (f), and *pacs-1*(R116W) (g). Adult animals showing localization

along ventral processes of GABAergic motor neurons. Imaged on Zeiss Axio Imager M2 compound microscope. Scale bar=10 μm. (h) Quantification of SNB-1::GFP puncta along dorsal (black) and ventral (gray) processes of adult GABAergic motor neurons. Numbers of puncta along dorsal or ventral processes are not significantly (ns) different for each genotype by unpaired t-tests ($p>0.1$).

Figure S6

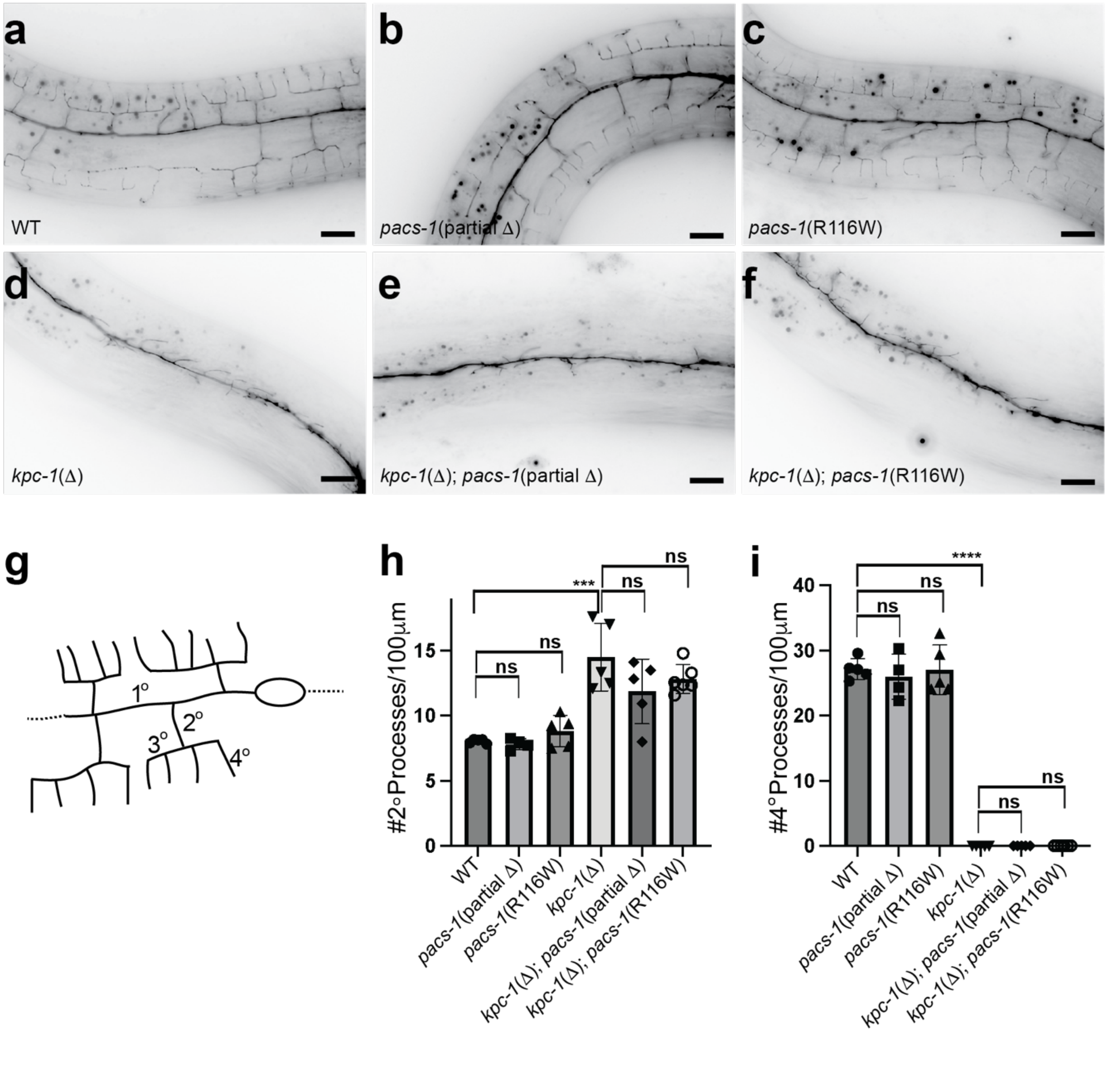

Figure S6: Analysis of PVD dendritic morphology in various *pacs-1* mutants.

(a-f) Structure of PVD neurons visualized with *wyIs378* showing dendritic branches in wild type (a), *pacs-1*(partial Δ) (*gk325*) (b) and *pacs-1*(R116W) (*ju1873*) (c) animals, and reduced dendritic structure in *kpc-1*(Δ) (*gk8*) (d), *kpc-1*(Δ) (*gk8*); *pacs-1*(partial Δ) (*gk325*) (g) and *kpc-1*(Δ) (*gk8*);

pacs-1(R116W) (*ju1873*) (f) animals. Images are composites from stack of 3 image planes taken on Zeiss AxioImager compound microscope. Animals oriented with anterior side to left. Scale bars=10μm. (g) Schematic of dendritic structure of PVD neuron with primary (1°), secondary (2°), tertiary (3°), and quaternary (4°) dendritic branches labeled. (h-i) Quantification of number of 2° branches (h) and 4° branches (i) per 100μM anterior of PVD cell body. Asterisks show results of unpaired t-tests where ***$p \leq 0.001$, ****$p \leq 0.0001$, ns=not significant. $n \geq 4$ for each genotype.

Table S1: Strains used in this study

strain	genotype	figure
PHX2274	*pacs-1*::GFP(*syb2274*) V	SunyBiotech; *pacs-1*::*gfp* in Figure 1a-c; 2c,f,i; 3c-d; S1d; S2a; S3a
CZ28192	*pacs-1*::GFP(*ju1822* R116W) V	*pacs-1* (R116W)::*gfp* in Figure 1d-f; 3g-h; S1f; S2a;
CZ29995	*pacs-1*::GFP(*ju1999* E205K) V	*pacs-1* (E205K)::*gfp* in Figure 1g-I; S1f
CZ30225	PACS-1(R116W)::GFP(*ju2021*) V	*pacs-1* (R116W E205K)::*gfp* in Figure 1j-l; S1f
N2	+	*RRID:CGC_N2*; Figures 2a,d,g*; S4a*
CZ29655	*pacs-1* (*ju1966*) V *pacs-1*::GFP(*syb2274*) V	*pacs-1* (Δ FBR)::*gfp* in Figure 2b,e,h; S1e; S2a; S4d
CZ28507	*wdr-37* (*ju1847*) III	*wdr-37* (Δ) in Figure 3b; S4f
PHX5283	*wdr-37*::mNG::3xFlag(*syb5283*) III	Figure 3b; 4a-b
CZ28510	*wdr-37* (*ju1847*) III ; *pacs-1*::GFP(*syb2274*) V	Figure 3e-f; S2b
CZ28511	*wdr-37* (*ju1847*) III ; *pacs-1*::GFP(*ju1822* R116W) V	Figure 3i-j; S2b; S4g
CZ29225	*wdr-37* (*ju1847*) III ; *pacs-1*::GFP(*syb2274*) V ; P*rgef-1*-WDR-37A cDNA(*juEx8178*)	Figure 3k
CZ29226	*wdr-37* (*ju1847*) III ; *pacs-1*::GFP(*syb2274*) V ; P*rgef-1*-WDR-37A cDNA(*juEx8179*)	Additional array for Figure 3k
CZ29227	*wdr-37* (*ju1847*) III ; *pacs-1*::GFP(*syb2274*) V ; P*rgef-1*-WDR-37A cDNA(*juEx8180*)	Additional array for Figure 3k
CZ29023	*wdr-37*::mNG::3xFlag(*syb5283*) III ; *pacs-1*::wrmScarlet(*syb2272*) V	Figure 4c top
CZ29024	*wdr-37*::mNG::3xFlag(*syb5283*) III ; *pacs-1* (*ju1827*) V *pacs-1*::wrmScarlet(*syb2272*) V	Figure 4c bottom
CZ30286	*wdr-37*::mNG::3xFlag(*syb5283*) III ; *pacs-1* (*ju2014*) V *pacs-1*::GFP(*syb2274*) V	Figure 4d
CZ30591	*wdr-37*::mNG::3xFlag (*syb5283*) III; *pacs-1* (*ju2014*) V; P*rgef-1*-*pacs-1* (*juEx8374*)	Figure 4e
CZ30592	*wdr-37*::mNG::3xFlag (*syb5283*) III; *pacs-1* (*ju2014*) V; P*rgef-1*-*pacs-1* (juEx8375)	Additional array for Figure 4e
C730593	*wdr-37*::mNG::3xFlag (*syb5283*) III; *pacs-1* (*ju2014*) V; P*rgef-1*-*pacs-1* (*juEx8376*)	Additional array for Figure 4e
CZ30594	*wdr-37*::mNG::3xFlag (*syb5283*) III; *pacs-1* (*ju2014*) V; P*rgef-1*-*pacs-1* (*juEx8377*)	Additional array for Figure 4e
C730595	*wdr-37*::mNG::3xFlag (*syb5283*) III; *pacs-1* (*ju2014*) V; *juEx8378*	Co-injection marker control for Figure 4e-f
CZ30596	*wdr-37*::mNG::3xFlag (*syb5283*) III; *pacs-1* (*ju2014*) V; *juEx8379*	Co-injection marker control for Figure 4e-f
C730597	*wdr-37*::mNG::3xFlag (*syb5283*) III; *pacs-1* (*ju2014*) V; *juEx8380*	Co-injection marker control for Figure 4e-f
CZ30600	*wdr-37*::mNG::3xFlag (*syb5283*) III; *pacs-1* (*ju2014*) V; P*rgef-1* -hPACS1 (WT) (*juEx8383*)	Figure 4f

CZ30598	*wdr-37*::mNG::3xFlag (*syb5283*) III; pacs-1 (*ju2014*) V; P*rgef-1* -hPACS1 (WT) (*juEx8381*)	Additional array for Figure 4f
C730599	*wdr-37*::mNG::3xFlag (*syb5283*) III; *pacs-1* (*ju2014*) V; P*rgef-1* -hPACS1 (WT) (*juEx8382*)	Additional array for Figure 4f
C730601	*wdr-37*::mNG::3xFlag (*syb5283*) III; *pacs-1* (*ju2014*) V; P*rgef-1* -hPACS1 (WT) (*juEx8384*)	Additional array for Figure 4f
CZ30214	*pacs-1* (*ju2014*) V *pacs-1*::GFP(*syb2274*) V	*pacs-1* (Δ) in Figure S1e; S2a; S4e
VC748	*pacs-1* (*gk325*) V	*pacs-1* (partial Δ) in Figure S1e, S2a; S3b-e; S4c
CZ28733	*pacs-1* (*ju1873*) V	*pacs-1* (R116W) in Figure S1f; S2a; S3d-e; S4b
PHX2272	*pacs-1*::wrmScarlet(*syb2272*) V	SunyBiotech; *pacs-1*::*mSc* in Figure S1i
CZ28197	*pacs-1* (*ju1827*) V *pacs-1*::wrmScarlet(*syb2272*) V	*pacs-1* (R116W)::*mSc* in Figure S1i; S3c
KP1097	*dgk-1* (*nu62*) X	(Nurrish et al. 1999); *dgk-1* (lf) in Figure S3d
CZ29432	*pacs-1* (*gk325*) V ; *dgk-1* (*nu62*) X	*pacs-1* (partial Δ); *dgk-1* (lf) in Figure S3d
CZ29433	*pacs-1* (*ju1873*) V ; *dgk-1* (*nu62*) X	pacs-1(R116W); *dgk-1* (lf) in Figure S3d
MT6242	*acr-2* (*n2420*) X	(Jospin et al. 2009); *acr-2* (gf) in Figure S3e
CZ28878	*pacs-1* (*gk325*) V ; *acr-2* (*n2420*) X	*pacs-1* (partial Δ); *acr-2* (gf) in Figure S3e
CZ28879	*pacs-1* (*ju1873*) V ; *acr-2* (*n2420*) X	*pacs-1* (R116W); *acr-2* (gf) in Figure S3e
CZ30498	*wdr-37* (*ju1847*) III ; *pacs-1* (*ju2014*) V	*wdr-37* (Δ);*pacs-1* (Δ) in Figure S4h
CZ10969	*mec-7*-GFP(*muIs32*) II	WT control for ALM morphology marker; Figure S5a
CZ16131	*mec-7*-GFP(*muIs32*) II ; *pacs-1* (*gk325*) V	Figure S5b
CZ29100	*mec-7*-GFP(*muIs32*) II ; *pacs-1* (*ju1873*) V	Figure S5c
CZ333	P*unc-25*-SNB::GFP(*juIs1*) IV	WT control for motor neuron synapse marker; Figure S5e
CZ28737	P*unc-25*-SNB::GFP(*juIs1*) IV ; *pacs-1* (*gk325*) V	Figure S5f
CZ28736	P*unc-25*-SNB::GFP(*juIs1*) IV ; *pacs-1* (*ju1873*) V	Figure S5g
TV12498	*ser-2prom3*::myr-GFP+P*rab-3*::myr-mCherry(*wyIs378*) X	(Wei et al. 2015); WT in Figure S6a
CZ29585	*pacs-1* (*gk325*) V ; *ser-2prom3*::myr-GFP+P*rab-3*::myr-mCherry(*wyIs378*) X	*pacs-1* (partial Δ) in Figure S6b
CZ29584	*pacs-1* (*ju1873*) V ; *ser-2prom3*::myr-GFP+P*rab-3*::myr-mCherry(*wyIs378*) X	*pacs-1* (R116W) in Figure S6c
VC48	*kpc-1* (*gk8*) I	(Thacker and Rose 2000; Schroeder et al. 2013)
CZ29586	*kpc-1* (*gk8*) I ; *ser-2prom3*::myr-GFP+P*rab-3*::myr-mCherry(*wyIs378*) X	*kpc-1* (Δ) in Figure S6d

CZ29588	*kpc-1* (*gk8*) I ; *pacs-1* (*gk325*) V ; *ser-2prom3* ::myr-GFP+P*rab-3* ::myr-mCherry(*wyIs378*) X	*kpc-1* (Δ); *pacs-1* (partial Δ) in Figure S6e
CZ29587	*kpc-1* (*gk8*) I ; *pacs-1* (*ju1873*) V ; *ser-2prom3* ::myr-GFP+P*rab-3* ::myr-mCherry(*wyIs378*) X	*kpc-1* (Δ); *pacs-1* (R116W) in Figure S6f

Table S2 - crRNA and oligo sequences

crRNAs

name	notes	IDT label	sequence
SD20246	*pacs-1* deletion alleles (*ju2014* and *ju1966*)	Ce.Cas9.TAG-232.1.AA	CCATGCGGTTGTATGCCACA
SD20670	*pacs-1* (R116W) edits and deletion alleles (*ju2014*)	CD.Cas9.GVFP9457.BG	TTTAAAAATCGTCTTCCTGG
SD20144	*pacs-1* FBR deletion allele (*ju1966*)	Ce.Cas9.TAG-232.1.AC	AGGTGCTTGTGAATAACAGG
SD21142	*pacs-1* (E205K) edits	Ce.Cas9.TAG-232.1.AW	GAAATGCCAAGTGCACGAAG
SD21143	*wdr-37* deletion allele (*ju1847*)	Ce.Cas9.C05D2.10.1.AF	AAGGTGATGCGTTCACGACG
SD21144	*wdr-37* deletion allele (*ju1847*)	Ce.Cas9.C05D2.10.1.AX	CACTTTCCAATGACCAGATA
SD21145	*wdr-37* deletion allele (*ju1847*)	CD.Cas9.VVJB5984.AB	GACCATTCTATCGAAACCAG
SD21149	*unc-58* co-CRISPR	CD.Cas9.DLJL7475.AA	ATCCACGCACATGGTCACTA
SD21045	*dpy-10* co-CRISPR	KO dpy-10 crRNA	CTACCATAGGCACCACGAG

CRISPR Repair Oligos

name	notes	IDT label	sequence
SD21146	*pacs-1* (R116W) Repair Oligo	PACS1repair	AATCAAATGTGCTTCAAATATTGATTCAACGAAGGAAAAAGTTTAAAAATtGgCTTCCTGGAGGTCTTCGTGATCTCGCTGTCGGGAATATTAATCTCACTTA
SD21147	*pacs-1* (E205K) Repair Oligo	cePACS2variantRepair	AAGATTCTGAAGATGAAACAGAAACAGACTATGATGATGTTGGTGATGAGAAGAATGAAATGCCAAGTGCtCGtAGCGGTCGGCATAAAACATCCACAGATAT
SD21148	*unc-58* co-CRISPR Repair Oligo	unc-58 repair oligo	ATTTTGTGGTATAAAATAGCCGAGTTAGGAAACAAATTTTTCTTTCAGGTTTCTCAGTAGTGACCATGTGCGTGGATCTTGCGTCCACACATCTCAAGGCGTACTT

Genotyping Oligos

name	notes	IDT label	sequence
JYS01-seq-s1	*pacs-1* (*syb2272* and *syb2274*) - forward	JYS01-seq-s1	ATCCCGTTCCGCAATCAT
JYS01-seq-a1	*pacs-1* (*syb2272* and *syb2274*) - reverse	JYS01-seq-a1	AAGCGTTTGTTGGTGCGAT
SD20229	*pacs-1* (E205K alleles) - forward	oCH_42	gCTCTCCAAACGAAATAGATATTTGCGG

SD20230	*pacs-1* (*ju2014* , *ju1966* , and E205K alleles) - reverse	oCH_43	CATTTCTTTGACCACCGGACGTCG
SD20247	*pacs-1* (*ju2014* , *ju1966*) - forward	oCH_46	caccggcggttgttctctaaac
YJ8603	*pacs-1* (*gk325*) - reverse	YJ8603	CGATTTATGGGCCAGACACT
YJ8604	*pacs-1* (*gk325*) - forward	YJ8604	TGACGACATCGATTCACCAT
SD20406	*pacs-1* PCR product for sequencing - forward	PACS1_5b	GTCGATAGAGCTACACCGTC
SD20407	*pacs-1* PCR product for sequencing - reverse	PACS1_3b	CTTCTGATCGATGCGTCATC
SD20127	*pacs-1* sequencing primer - forward	PACS1_5c	GCAACGAGATAAAGGTGACG
SD20128	*pacs-1* sequencing primer - reverse	PACS1_3c	ACCACCTTGTTGCATTATCTG
SD20271	*pacs-1* (R116W) variant detection PCR - forward	pacs1common5.2	GCTACACCGTCTACTGTTCCGAG
SD20272	*pacs-1* (WT) variant detection PCR - reverse	pacs1wt3.1	CACGAAGACCTCCAGGAAGaag
SD20273	*pacs-1* (R116W) variant detection PCR - reverse	pacs1mut3.1	CACGAAGACCTCCAGGAAGcaa
SD20275	*unc-58* control PCR and co-CRISPR sequencing - forward	unc58seq5.1	CGGACTCGGAGATATCGTTGTG
SD20274	*unc-58* control PCR and co-CRISPR sequencing - reverse	unc58seq3.1	GCTTCATCAGAAGCTCATGAGG
SD20437	*wdr-37* (*syb5283*) - forward	oCH_20	CACGAACGAATGGTGACGTG
SD20438	*wdr-37* (*syb5283*) - reverse	oCH_21	ggaatgtctcatggctaaaatgtc
SD20315	*wdr-37* (*ju1847*) - forward	wdr37_1.5	cacacgtggaaattgatccg
SD20316	*wdr-37* (WT) - reverse	wdr37_3.3	CGGATCCTTCAGTACTATCCAC
SD20317	*wdr-37* (*ju1847*) - reverse	wdr37_4.3	ttctgaagcaatcgctcgtc

RT PCR Oligos

**name**	**notes**	**IDT label**	**sequence**
SD20344	*pacs-1* cDNA exon 6	oCH_48	CGACGTCCGGTGGTCAAAGAAATG
SD20346	*pacs-1* cDNA exon 8	oCH_50	GATTGACGAGATTGGCGAGCG

SD20382	*mdt-27* cDNA - forward	oCH_51	GGGAGACAATACAATCCGGAT
SD20383	*mdt-27* cDNA - reverse	oCH_52	CTCTCGTCCATTGCCTCTTG
SD20368	*act-1* cDNA - forward	act1_fwd	GTTGCCCAGAGGCTATGTTC
SD20369	*act-1* cDNA - reverse	act1_rev	CAAGAGCGGTGATTTCCTTC

Cloning Oligos

name	notes	IDT label	sequence
SD20414	*pacs-1* cDNA - forward	OCP333F	ATGGATAAACAACATGAGAGC
SD20415	*pacs-1* cDNA - reverse	OCP334R	TCAAGCTGTGCAGACATTCGG
SD20416	*wdr-37* cDNA - forward	oCH_8	ATGCCACAATCGGAGAATTC
SD20417	*wdr-37* cDNA - reverse	oCH_9	TTATGAAGTTGTTGTATTGACTT TCCACGC
SD20425	To verify Gateway cloning into pCZGY66 - forward	oCH_18	ccaacgttctttctagatcttgagac
SD20426	To verify Gateway cloning into pCZGY66 - reverse	oCH_19	CAGGGAGAAAGAGCATGTAGG G
SD21150	To verify Gateway cloning of hPACS1 cDNA (colony PCR) - forward	hPACS1_2F	CCTGAAGAAACTCGTCATGCT
SD21151	To verify Gateway cloning of hPACS1 cDNA (colony PCR) - reverse	hPACS1_2R	GTGATGGCAGAGGTTGAGGT
SD21152	To verify Gateway cloning of hPACS1 cDNA (sequence of promoter junction)	hPACS1_Sanger_1_R	GATCTCGTTGGAGCGAAGAA

Table S3 - DNA expression constructs

name	description	extrachromosomal arrays	microinjection info
pCZGY3615	P*rgef-1*-*wdr37* cDNA	*juEx8178 juEx8179 juEx8180*	Injected at 10ng/uL with 30ng/uL of *rol-6* (gf)
pCZGY3630	P*rgef-1*-*pacs-1* cDNA	*juEx8374*, *juEx8375*, *juEx8376*, *juEx8377*	Injected at 50 ng/uL with 50 ng/uL P*ttx-3*-dsRed
pCZGY3631	P*rgef-1*-hPACS1 cDNA	*juEx8381*, *juEx8382*, *juEx8383*, *juEx8384*	Injected at 50 ng/uL with 50 ng/uL P*ttx-3*-dsRed
